# Unusual thalamic mass and subsequent gelatinous pseudocysts in an immunocompetent host: A case report

**DOI:** 10.1016/j.idcr.2022.e01602

**Published:** 2022-08-17

**Authors:** Apapatra Akiko Watanabe, Pasin Hemachudha, Wanakorn Rattanawong, Thanakit Pongpitakmetha

**Affiliations:** a Faculty of Medicine, Chulalongkorn University, Bangkok 10330, Thailand; b Department of Medicine, Panyananthaphikkhu Chonprathan Medical Center, Srinakharinwirot University, Nonthaburi 11120, Thailand; c Thai Red Cross Emerging Infectious Diseases Health Science Centre, World Health Organization Collaborating Centre for Research and Training on Viral Zoonoses, King Chulalongkorn Memorial Hospital, Faculty of Medicine, Chulalongkorn University, Bangkok 10330, Thailand; d Division of Neurology, Department of Medicine, Chulalongkorn University, King Chulalongkorn Memorial Hospital, Faculty of Medicine, Chulalongkorn University, Bangkok 10330, Thailand; e Department of Medicine, Faculty of Medicine, King Mongkut's Institute of Technology Ladkrabang, Bangkok, Thailand; f Department of Pharmacology, Faculty of Medicine, Chulalongkorn University, Bangkok 10330, Thailand; g Chula Neuroscience Center, King Chulalongkorn Memorial Hospital, Faculty of Medicine, Chulalongkorn University, Bangkok 10330, Thailand

**Keywords:** Cryptococcus, Gelatinous pseudocyst, Meningoencephalitis, Case report

## Abstract

Cryptococcal meningoencephalitis often occurs in an immunocompromised host with several known neurological manifestations including space-occupying lesions, meningitis or meningoencephalitis. Here, we describe a 38-year-old previously healthy durian farm owner with cryptococcoma and subsequent development of cryptococcus gelatinous pseudocyst after receiving high doses of intravenous dexamethasone to treat mass lesion presumed to be a malignant process. An MRI scan of the head demonstrated a 2-cm heterogeneous solitary enhancing cystic lesion at the right thalamus. Progression of neurological deficit and another repeat imaging showing typical appearance of gelatinous pseudocyst. Lumbar puncture found markedly elevated pressure and cryptococcal antigen strongly positive confirming the diagnosis. He was immediately started on amphotericin B and flucytosine for cryptococcus meningoencephalitis with partial improvement in his vision. This report highlights consideration of cryptococcal infection in an immunocompetent host to avoid delays in diagnosis and treatment.

## Case presentation

**Fig. 1:**
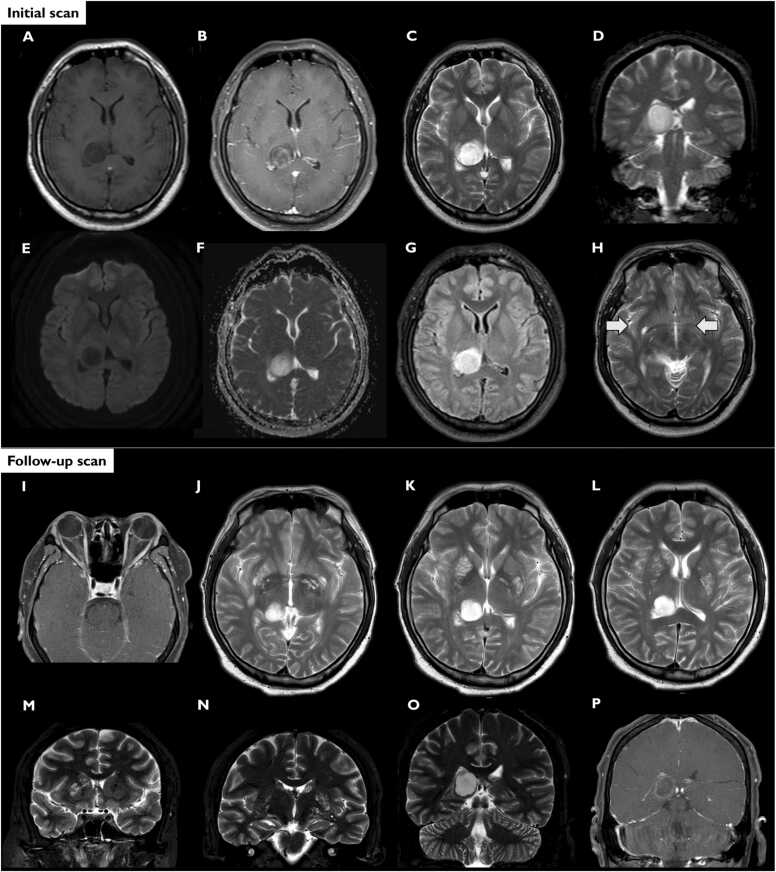
Initial and follow-up MRI brain. T1-weighted (A), T1-weighted with gadolinium (B), T2-weighted (C, D, H), diffusion-weighted images (DWI) (E), apparent diffusion coefficient (ADC) (F), and T2-fluid-attenuated inversion recovery (FLAIR) (G).

A 38-year-old male durian farm owner in Thailand with no previous medical history developed reduced visual acuity in both of his eyes for 3 days before presenting to our hospital. It was preceded by a headache, which led him to his local hospital after a week. No neurological deficits were found at the time but further investigation with magnetic resonance imaging (MRI) of the brain showed a 2-cm heterogeneous enhancing cystic lesion at the right thalamus (Fig. 1). The provisional diagnosis was a brain tumour. He was hospitalised and received intravenous dexamethasone at 8 mg per day and pethidine for the pain. He later developed reduced vision of the left eye 8 days after receiving the treatment. Declining acuity of the right eye was noted over the following 2 days. He self-referred to our hospital for further management.

His initial examination showed diffuse erythematous umbilicated pustules. Neurological examination showed reduced visual acuity: on the right eye was a finger count at 1 foot with intact visual field and no light perception in the left eye. Both pupils were 5 millimetres and only slightly reactive to light with relative afferent pupillary reflex on the left. Fundoscopic examination was normal with no papilloedema. Resting eye position was in midline but with impaired eye movement in all directions in both eyes. Other cranial nerve examinations revealed right upper motor neuron facial weakness, reduced left gag reflex.

Another MRI of the brain was done 2 weeks after the previous MRI and showed a similar cystic lesion, probably cryptococcoma (Fig. 1). Other positive MRI findings were multiple enlarged perivascular spaces involving bilateral basal ganglia, probably gelatinous pseudocysts with leptomeningeal enhancement and abnormal peri-optic enhancement. CT scan of the chest with contrast showed a 4×3x2-cm well-defined, irregular-shaped, non-enhancing mass at the lateral segment of the right middle lobe.

**Fig. 2:**
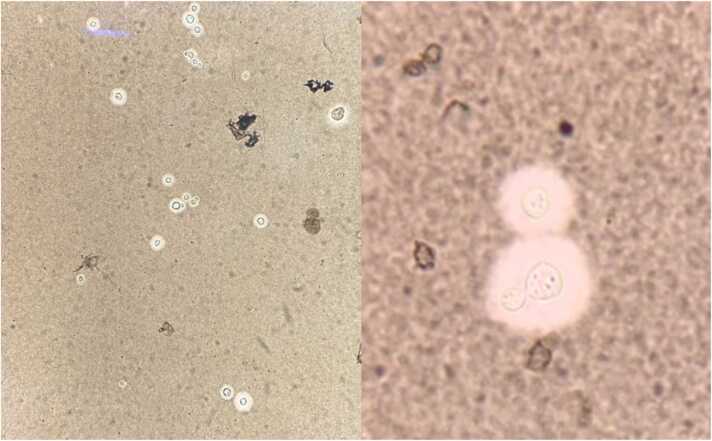
India ink preparation of the cerebrospinal fluid. At 40x magnification (Left) and 100x magnification (Right), showing encapsulated budding yeast cells and a few single yeast cells without budding morphology, demonstrating Cryptococcus. The polysaccharide capsule surrounding the cell results in the appearance of a halo around the cell under India ink preparation.

Lumbar puncture showed colourless CSF with opening pressure of 60 cmH_2_O, closing pressure of 40 cmH_2_O, WBC 56 cells/uL (Polymorphonuclear cell 8.9 %, Lymphocyte 91.1 %), RBC 0 cell/uL, protein 48.6 mg/dL, glucose 69 mg/dL (random blood sugar 151 mg/dL). Cryptococcal antigen was positive with a titer of 1:2048 and encapsulated budding yeast was seen from India ink examination (Fig. 2). CSF fungal culture revealed *Cryptococcus gattii*. Wright’s stain of a chest papule was also positive for budding yeast. His anti-HIV testing was negative and his immunoglobulin levels were within normal limits.

The diagnosis of cryptococcal meningoencephalitis with disseminated *Cryptococcus gattii* was established and he was started on Amphotericin B 0.75 mg/kg/day with flucytosine 12 g/day and underwent surgery for a ventriculoperitoneal shunt. His visual acuity and eye movement improved partially after 10 days of treatment initiation, and he was transferred back to his local hospital to complete his treatment.

## Discussion

Cryptococcus species capable of infecting humans include *C. neoformans* and *C. gattii* and can be found worldwide. *C. neoformans* is generally found in soil contaminated by pigeon droppings. While *C. gattii* is mostly found in tropical and subtropical regions [1], [2]. Eucalyptus trees are considered a natural habitat for *C. gattii;* in fact, our patient’s durian field was previously occupied by eucalyptus trees [3].

*C. gattii* has a significantly higher prevalence amongst immunocompetent hosts than *C. neoformans* and infection commonly occurs in the pulmonary system [1], [4], [5]. Time from symptom onset to diagnosis ranges from 45 to 52 days, longer when compared to *C. neoformans* possibly due to rarity and lower clinical suspicion [1], [5]. *C. gattii* infections are more likely to have longer hospitalization periods, need higher doses of antifungal therapy and result in more sequelae [4].

Common clinical manifestations of *C. gattii* infection of the CNS include headache, night sweats, weight loss, anorexia, and neck stiffness [4], [6]. Other neurological symptoms include impaired consciousness, cerebellar deficits, limb weakness, seizures and cranial nerve deficits [5]. Complications arising from the infection include elevated intracranial pressure, formation of cryptococcomas, and cerebral infarction. Infarction is thought to be directly related to local pressure effects or indirectly through vasculitis processes [7]. Another complication more frequently occurs in *C. gattii* infection is visual impairment, suggesting a predilection of *C. gattii* for visual pathway [7].

Cryptococcal abilities to evade host immune responses are through several mechanisms. Structurally, its encapsulation protects against oxidative damage induced by the host’s macrophages [7]. *C. gattii* can also suppress dendritic cell maturation causing impairment in the antigen-presenting processes [8]. It primarily infects the lung. If left unchecked by the immune system, it may disseminate hematogenously to various organs with a predilection to the CNS. Cryptococcus can cross the blood-brain barrier (BBB) via receptor-mediated transcytosis or secondary to leaky membranes. Cryptococcus is then carried through infected phagocytes to cause meningitis or a formation of cryptococcoma. Progression of infection is usually via the subarachnoid space and evading through perivascular space [7]. A histopathology report of a patient with cranial nerve involvement found infiltrations and axonal disruption without evidence of vasculitis. Rather than a direct invasion, mucinous exudates compressing perineural vascular plexus causing anoxic axonal injury was suspected [9].

Regarding imaging modalities, MRI of the brain has higher sensitivity for small lesions and basilar meningeal enhancement than CT. Cryptococcal meningoencephalitis can cause enlarged perivascular space, pseudocysts, cryptococcoma, leptomeningeal or parenchymal enhancing lesions. Enlarged perivascular space appears as punctate hyperintensities on T2-weighted images, resulting from disseminated infection and is commonly seen in basal ganglia, thalamus, midbrain and cerebellum, together with pseudocysts [10]. Cryptococcomas are more likely due to *C. gattii* and can easily be misdiagnosed as a brain tumour [1]. The common site for cryptococcoma includes the cerebellum, basal ganglia and thalamus [7]. Cryptococcomas typically present as solitary or multiple cystic lesions with low signal intensity on T1-weighted and high intensity on T2-weighted images. Diffusion-weighted images (DWI) and apparent diffusion coefficient (ADC) might be able to distinguish pyogenic brain abscess and cryptococcomas. Hypointensity on DWI and hyperintensity on ADC of the central cavity of a cryptococcomas was reported. While pus in pyogenic brain abscess gives restricted diffusion, a solid granuloma with a high signal mosaic pattern on DWI was also reported [11], [12]. Hazy brain base sign, a uniform and symmetrical hazy T2 signal increase of the basal brain such as basal ganglia, midbrain, thalamus and hypothalamus, is believed to be caused by the penetration of fungal material to the parenchyma and perivascular spaces. Additionally, polycranial neuritis can also be found, especially in HIV-negative patients [10].

Treatment in a non-HIV patient includes induction therapy of Amphotericin B plus flucytosine for six weeks, consolidation therapy with fluconazole for eight weeks, maintenance therapy with fluconazole for 6–12 months [7], [13]. Furthermore, one of the essential aspects of management is CSF pressure reduction. Serial lumbar punctures are recommended in symptomatic increased intracranial pressure and CSF pressure more or equal to 25 cmH_2_O [7], [13]. Half of the patients with hydrocephalus due to CSF outflow obstruction require a shunt or drainage if conservative method fails [5], [13].

## Conclusion

The diagnosis of cryptococcal CNS infection is often delayed in an immunocompetent individual. Early consideration of cryptococcal is important as MRI findings of cryptococcoma are commonly misdiagnosed as brain tumours. Findings of multiple enlarged perivascular spaces involving bilateral basal ganglia should also prompt strong suspicion of gelatinous pseudocysts found in cryptococcal meningoencephalitis demonstrated in this case.

### Initial scan

A 2-cm heterogeneous enhancing cystic lesion at the right dorsomedial thalamus (B) with hypointense T1 (A), hyperintense T2 (C, D), and some perilesional edema (F). T2-weighted sequence (C, D), DWI, (E), and ADC (F) showed increased diffusion pattern. Multiple enlarged perivascular spaces (Arrow in H) involve deep white matter of bilateral cerebral hemispheres, probably caused by gelatinous pseudocysts.

### Follow-up scan

A T1-weighted with gadolinium (I) showed an abnormal perioptic enhancement along bilateral intraorbital optic nerve sheaths and the retrobulbar regions. An approximately 2-cm well-defined rim-enhancing cystic lesion in the right dorsomedial thalamus, probably cryptococcoma, as well as the leptomeningeal enhancement with smooth enhanced dural thickening were also detected more pronounce in the posterior falx cerebri and parieto-occipital regions in a T1-weighted sequence with gadolinium (P).

In the axial and coronal T2 weighted showed a 2 cm cystic lesion in the right dorsomedial thalamus and multiple enlarged perivascular spaces involving bilateral basal ganglia, probably gelatinous pseudocysts (J-O).

## Ethical approval

Our institution does not require ethical approval for reporting individual cases or case series.

## Consent to participate

Our institution does not require ethical approval for reporting individual cases or case series and consent for participation in the case report have been obtained from the patient.

## Consent to publish

Written informed consent was obtained from the patient for anonymized patient information to be published in this article.

## Funding

The authors received no financial support for the article’s research, authorship, and publication.

## Author contributions

AAW and PH acquired the data; coordinated imaging; designed and conceptualized case; and drafted the manuscript for intellectual content. TP acquisition data; coordinated imaging; designed and conceptualized case; revised the manuscript for intellectual content. WR designed and conceptualized case; revised the manuscript for intellectual content.

## Declaration of Conflict of Interest

The authors report no relevant disclosures or conflict of interest.
